# Therapeutic Effect of MG132 on the Aortic Oxidative Damage and Inflammatory Response in OVE26 Type 1 Diabetic Mice

**DOI:** 10.1155/2013/879516

**Published:** 2013-03-26

**Authors:** Xiao Miao, Wenpeng Cui, Weixia Sun, Ying Xin, Bo Wang, Yi Tan, Lu Cai, Lining Miao, Yaowen Fu, Guanfang Su, Yuehui Wang

**Affiliations:** ^1^The Second Hospital of Jilin University, Changchun 130041, China; ^2^KCHRI, Department of Pediatrics, University of Louisville, Louisville, KY 40202, USA; ^3^Department of Ophthalmology, The Second Hospital of Jilin University, 218 Ziqiang Street, Changchun 130041, China; ^4^The First Hospital of Jilin University, Changchun 130021, China; ^5^Normal Bethune Medical College of Jilin University, Changchun 130021, China; ^6^Department of Pathology, Inner Mongolia Forestry General Hospital, Yakeshi, Inner Mongolia 022150, China; ^7^Chinese American Research Institute for Diabetic Complications, Wenzhou Medical College, Wenzhou 325000, China; ^8^Departments of Radiation Oncology and Pharmacology and Toxicology, University of Louisville, Louisville, KY 40202, USA; ^9^Department of Cardiology, The Second Hospital of Jilin University, 218 Ziqiang Street, Changchun 130041, China

## Abstract

The present study tested whether MG132 increases vascular nuclear factor E2-related factor-2 (Nrf2) expression and transcription to provide a therapeutic effect on diabetes-induced pathogenic changes in the aorta. To this end, three-month-old OVE26 diabetic and age-matched control mice were intraperitoneally injected with MG-132, 10 **μ**g/kg daily for 3 months. OVE26 transgenic type 1 diabetic mice develop hyperglycemia at 2-3 weeks of age and exhibit albuminuria at 3 months of age with mild increases in TNF-**α** expression and 3-NT accumulation in the aorta. Diabetes-induced significant increases in the wall thickness and structural derangement of aorta were found in OVE26 mice with significant increases in aortic oxidative and nitrosative damage, inflammation, and remodeling at 6 months of diabetes, but not at 3 months of diabetes. However, these pathological changes seen at the 6 months of diabetes were abolished in OVE26 mice treated with MG-132 for 3 months that were also associated with a significant increase in Nrf2 expression in the aorta as well as transcription of downstream genes. These results suggest that chronic treatment with low-dose MG132 can afford an effective therapy for diabetes-induced pathogenic changes in the aorta, which is associated with the increased Nrf2 expression and transcription.

## 1. Introduction

Diabetes mellitus is a chronic disease that affects many people worldwide now. There are two major types of diabetes mellitus: type 1 (insulin dependent) diabetes and type 2 (noninsulin dependent) diabetes. Systemic complications are major cause of the mortality in these patients with either type 1 or type 2 diabetes. These complications are divided into two groups: microvascular complications, which include retinopathy, nephropathy, and neuropathy; and macrovascular complications, which include coronary artery disease, atherosclerosis, and peripheral vascular disease.

Increasing evidence indicates that increased production of reactive oxygen or nitrogen species (ROS or RNS) and/or impaired endogenous protective mechanisms is the major risk factor responsible for the development and progression of vascular complications in diabetic patients, although several other mechanisms were also proposed [1, 2]. Exogenous supplementation of a single or a few antioxidants in clinics often fails to efficiently prevent or treat various complications for diabetic patients; therefore upregulation of multiple, endogenous antioxidants may be a better approach for the prevention of diabetic cardiovascular complications.

Nuclear factor E2-related factor-2 (Nrf2), as a transcription factor in regulation of various antioxidative and cyto-protective responses to oxidative stress, has been shown to play an important role in cellular prevention against oxidative stress and damage *in vitro* and *in vivo* [3, 4]. Actin-tethered protein Keap1 is a cytosolic repressor that binds to and retains Nrf2 in the cytoplasm, which promotes Nrf2 proteasomal degradation so as to prevent Nrf2 activation of its transcription [3, 4]. Via blocking Nrf2 proteasomal degradation, therefore, proteasome inhibitors are able to retain Nrf2 in cytoplasm and/or to translocate into nuclei. Therefore, proteasomal inhibitors have become an attractive approach to activate Nrf2-mediated anti-oxidative pathway in the prevention of various oxidative stress-initiated diseases [5]. Among proteasome inhibitors, MG132 is specific, potent, reversible, and cell permeable and plays a key role in blocking the degradation of ubiquitin-conjugated proteins in mammalian cells by the 26S complex without affecting its ATPase or isopeptidase activities [6].

Reportedly, up-regulation of Nrf2 by its activators, including MG132, has been found to potentially be a preventive approach for diabetic complications [5, 7–9], including diabetes-induced vascular injuries [10–13]. However, there was no report yet whether, upregulating Nrf2 can also efficiently cure or slow the progression of established diabetic complications.

Therefore, the present study was to examine the therapeutic effect of chronic treatment with low-dose MG132 on the pathogenic changes of aortas in the transgenic (OVE26) diabetic mouse model. The treatment was started when OVE26 mice exhibited significant increase in albuminuria at 3 months of age. The expression of Nrf2 and its downstream antioxidant genes were examined. We found that the progression of aortic pathogenic change was almost completely stopped by 3-month treatment with MG132, which was associated with a significant upregulated expression of Nrf2 and its downstream antioxidant genes.

## 2. Methods

### 2.1. Animals

OVE26 type 1 diabetic mice with FVB background were generated and provided for this study by Epstein et al. [14, 15]. Mice were housed in the University of Louisville Research Resources Center at 22°C with a 12-h light/dark cycle and provided with free access to standard rodent chow and tap water. All animal procedures were approved by the Institutional Animal Care and Use Committee, which is certified by the American Association for Accreditation of Laboratory Animal Care.

These OVE26 mice normally develop severe hyperglycemia 2-3 weeks after birth and develop macroproteinuria significantly at 3 months of age [14, 15]. Sixteen 3-month-old female OVE26 mice were randomly divided into two groups: diabetes (DM, *n* = 10) and diabetes treated with MG132 (DM/MG132, *n* = 6). Sixteen age- and sex-matched wild-type (FVB) mice were also randomly divided into two groups: nondiabetic control (control, *n* = 10) and MG132 control (MG132, *n* = 6). MG132 (Sigma-Aldich, St. Louis, MO, USA) was dissolved in dimethyl sulfoxide (DMSO) at a concentration of 0.0025 *μ*g/mL. For MG132 and DM/MG132 mice, MG132 was given intraperitoneally at 10 *μ*g/kg daily for 3 months, while control and DM group mice were administered with equal amounts of MG132 vehicle. The dose of MG132 was selected based on a recent study [16], which is significantly lower than that used in other studies to efficiently protect the heart from oxidative damage [17, 18]. Four mice from both control and DM group were sacrificed at 3 months of age, and the rest (6 mice in each group) were sacrificed at the end of 3-month treatment with MG132 (i.e., at 6 months of age).

### 2.2. Noninvasive Blood Pressure

Blood pressure (BP) was measured by tail-cuff manometry using a CODATM noninvasive BP monitoring system (Kent Scientific, Torrington, CT, USA). Mice were restrained in a plastic tube restrainer. Occlusion and volume-pressure recording (VPR) cuffs were placed over the tail. Mice were allowed to adapt to the restrainer for 5 min prior to starting BP measurement. After a 5 min adaptation period, BP was measured for 10 acclimation cycles followed by 20 measurement cycles. Mice were warmed by heating pads during the acclimation cycles to ensure sufficient blood flow to the tail. Animals were monitored closely throughout the measurement protocol and removed from restraint as soon as possible upon completing the measurement protocol [19]. After three days of training for the BP measurement, formal measurements were performed and BP data were collected.

### 2.3. Aorta Preparation and Histopathological Examination

After anesthesia, thorax was opened, and descending thoracic aortas were isolated carefully and cleaned of the surrounding fat and connective tissue. Aortic tissues were fixed in 10% buffered formalin and then cut into ring segments (2-3 mm in length) for being dehydrated in graded alcohol series, cleared with xylene, embedded in paraffin, and sectioned at 5 *μ*m thickness in order to perform pathological and immunohistochemical or immunofluorescent examination, as described in previous study [20]. Histological evaluation of aorta was performed by H&E staining with Image Pro Plus 6.0 software for measuring the means of the tunica media width size as the thickness of aortic tunica media.

For immunohistochemical or immunofluorescent staining, paraffin sections were dewaxed, followed by incubation with 1X Target Retrieval Solution (Dako, Carpinteria, CA, USA) in a microwave oven for 15 min at 98°C for antigen retrieval, with 3% hydrogen peroxide for 15 min at room temperature, and then with 5% animal serum for 30 min, respectively. These sections were incubated with primary antibodies, including those against connective tissue growth factor (CTGF) and transforming growth factor (TGF)-*β*1 (Santa Cruz Biotechnology, Santa Cruz, CA, USA) at 1 : 100 dilution, 3-nitrotyrosine (3-NT, Millipore, Billerica, CA, USA) at 1 : 400 dilution, 4-hydroxy-2-nonenal (4-HNE, Alpha Diagnostic International, San Antonio, TX, USA) at 1 : 400 dilution, plasminogen activator inhibitor-1 (PAI-1, BD Bioscience, San Jose, CA, USA) at 1 : 100 dilution, tumor necrosis factor-alpha (TNF-*α*, Abcam, Cambridge, MA, USA) at 1 : 100 dilution, Cu-Zn superoxide dismutase-1 (SOD-1) at 1 : 400 dilution, and Nrf2 (both from Santa Cruz Biotechnology) at 1 : 100 dilution, respectively, overnight at 4°C. After sections were washed with PBS, they were incubated with horseradish peroxidase-conjugated secondary antibodies (1 : 300–400 dilutions with PBS) or Cy3-coupled goat antirabbit IgG secondary antibody for 2 h in room temperature. For the color development of immunohistochemical staining, sections were treated with peroxidase substrate DAB kit (Vector Laboratories, Inc., Burlingame, CA, USA) and counterstained with hematoxylin. For immunofluorescent staining, sections were stained with DAPI at 1 : 1000 dilution to localize the nucleus. Three sections at interval of 10 sections from each aorta (per mouse) were selected and at least five high-power fields were randomly captured per section. Image Pro Plus 6.0 software was used to transfer the interesting area staining density to an integrated optical density (IOD) that was divided by the area size of interest to reflect the staining level of the area of interest, and the ratio of IOD/area size in experimental group was presented as a fold relative to that of control.

### 2.4. Sirius-Red Staining for Collagen

Aortic fibrosis was detected by examining collagen accumulation with Sirius-red staining. Briefly, 5 *μ*m thickness sections were stained with 0.1% Sirius-red F3BA and 0.25% Fast Green FCF and then assessed for the proportion of collagen with a Nikon Eclipse E600 microscopy system [21].

### 2.5. Real-Time RT-PCR (qPCR)

Aortas were snap frozen in liquid nitrogen and kept at −80°C. Total RNA was extracted using the TRIzol reagent (Invitrogen). RNA concentrations and purities were quantified using a Nanodrop ND-1000 spectrophotometer. First-strand complimentary DNA (cDNA) was synthesized from total RNA, according to manufacturer's protocol from the RNA PCR kit (Promega, Madison, WI, USA). Reverse transcription system was performed using 1 *μ*g of total RNA in 12.5 *μ*L of the solution containing 4 *μ*L 25 mM MgCl_2_, 4 *μ*L AMV reverse transcriptase 5X buffer, 2 *μ*L dNTP, 0.5 *μ*L RNase inhibitor, 1 *μ*L of AMV reverse transcriptase, 1 *μ*L of oligo dT primer, and nuclease-free water that was added to make a final volume of 20 *μ*L. Reaction system was run at 42°C for 50 min and 95°C for 5 min, as described before [21]. 

Real-time RT-PCR (quantitative PCR, qPCR) was carried out with the ABI 7300 real-time PCR system in a 20 *μ*L reaction buffer, composed of 10 *μ*L of TaqMan Universal PCR Master Mix, 1 *μ*L of primer, and 9 *μ*L of cDNA. Primers of NADPH quinine oxidoreductase 1 (NQO1), heme oxygenase-1 (HO-1), SOD1, and *β*-actin were purchased from Applied Biosystems (Carlsbad, CA, USA). The fluorescence intensity of each sample was measured at each temperature change to monitor amplification of the target gene.

### 2.6. Statistical Analysis

Data collected from several animals (*n* = 4 for study at 3 months of age; *n* = 6 for study at 6 months of age) were presented as means ± SD. We used Image Pro Plus 6.0 software with an integrated optical density divided area method to identify the positive staining area of interest. Comparisons were performed by one-way ANOVA for the different groups, followed by post hoc pairwise repetitive comparisons using Tukey's test or Student's *t*-test with Origin 7.5 Lab data analysis and graphing software. Statistical significance was considered as *P* < 0.05.

## 3. Results

### 3.1. Pathogenic Changes in the Aorta of OVE26 Diabetic Mice at 3-Month-Old

**Figure 1:**
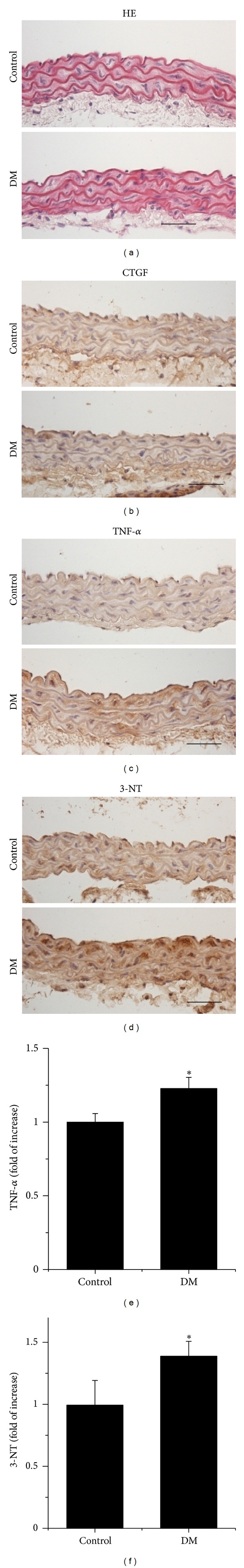
Diabetes-induced aortic pathological changes, inflammation, oxidative stress, fibrosis at 3 months by H&E staining (a), immunohistochemical staining for the expression of CTGF (b), TNF-*α* (c)-(e) as index of inflammation, and 3-NT (d)-(f) as an index of oxidative damage. Data were presented as means ± SD (*n* = 4). *P < 0.05 versus control. Bar = 50 *μ*M.

OVE26 mice develop diabetes before 3-week old and exhibit a significant increase in albuminuria at 3 months of age, as an index of renal dysfunction [15]. To define whether there was any pathological change in aortas of 3-month-old OVE26 diabetic mice, microscopic examination of aortas with H&E staining for the general morphology (Figure 1(a)) revealed that there was no significantly morphological abnormality, except for a slight derangement of endothelial and smooth muscle cells, in the aorta of OVE26 diabetic (DM) mice at 3 months of age. Immunohistochemical staining for CTGF as an important fibrotic mediator showed no significant difference between control and DM mice (Figure 1(b)).

However, there was an increase in TNF-*α* expression in the aorta of DM mice compared to control mice (Figures 1(c) and 1(e)) and also an increase in 3-NT accumulation, as an index of protein nitration, in the aorta of DM mice compared to control mice (Figures 1(d) and 1(f)).

### 3.2. Therapeutic Effects of MG132 on the Aortic Fibrotic Response

Previous results suggest a significant increase of aortic inflammation and oxidative stress and damage in 3-month-old DM mice compared to age-matched control mice, suggesting an induction of aortic early pathogenesis. To explore the therapeutic effect of MG132 on diabetes-induced progression of aortic pathological changes, we treated 3-month-old DM and age-matched control mice with low-dose MG132 for 3 months.

**Figure 2:**
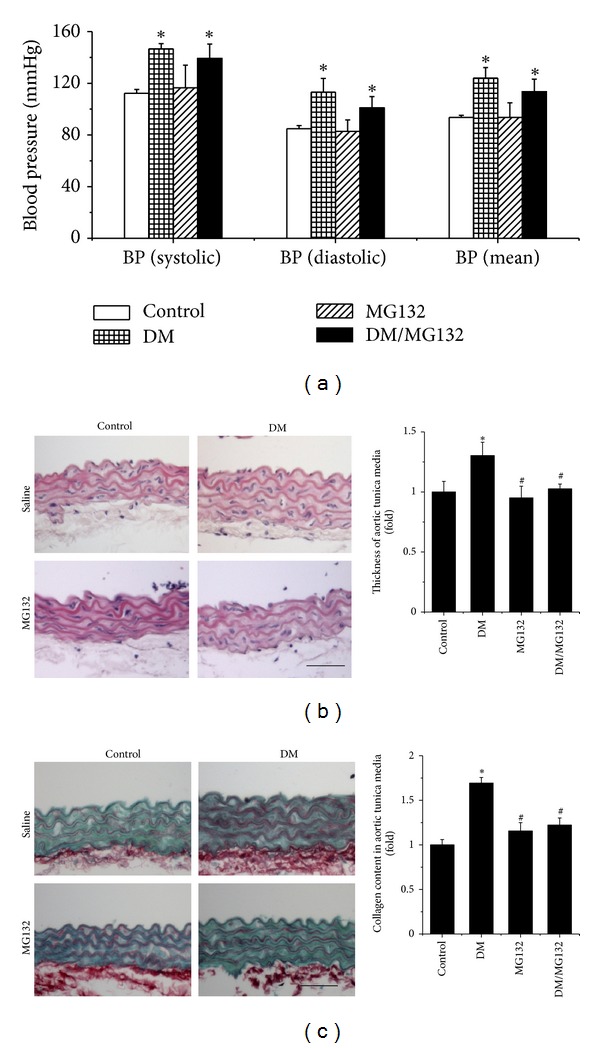
Therapeutic effect of MG132 on diabetes-induced blood pressure changes (a) and aortic pathological changes, examined by H&E staining (b), and Sirius-red staining for collagen accumulation (c) with semi-quantitative analysis. Data were presented as means ± SD (*n* = 6). *P < 0.05 versus control;  ^#^ *P* < 0.05 versus DM. Bar = 50 *μ*M.

Figure 2(a) showed no effect of chronic treatment with MG132 on systemic and diastolic blood pressure, although both were significantly higher in DM group than in control. H&E staining of the aortas showed that part of intima was thickened and uplifted; endothelial cells in the surface of intima were swelled and internal elastic membrane was thickened; tunica media is thickened. However, these changes observed in DM group were not observed in DM/MG132 group (Figure 2(b)). By Sirius-red staining, the collagen accumulation in the aorta was also only observed in DM group, but not in DM/MG132 group (Figure 2(c)).

**Figure 3:**
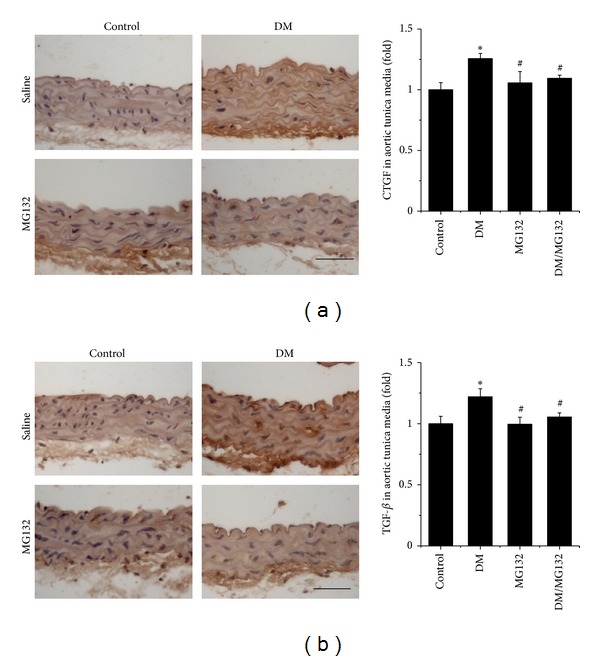
Therapeutic effect of MG132 on diabetes-induced aortic fibrosis by immunohistochemical staining for the expression of CTGF (a) and TGF-*β*1 (b) with semi-quantitative analysis. Data were presented as means ± SD (*n* = 6). *P < 0.05 versus control;  ^#^ *P* < 0.05 versus DM. Bar = 50 *μ*M.

Immunohistochemical staining showed that although there was no significant increase of the CTGF expression in the aorta of DM mice at 3 months of age compared to the age-matched control mice (Figure 1(b)), there was a significantly increased CTGF expression in the aorta of DM at 6 months of age compared to age-matched control mice (Figure 3(a)). Diabetes-increased CTGF expression was not seen in the aorta of DM/MG132 mice (Figure 3(a)). Similarly, aortic expression of another important profibrotic mediator, TGF-*β*1, was also observed only in the DM group and not in the DM/MG132 group (Figure 3(b)).

### 3.3. Therapeutic Effects of MG132 on Aortic Inflammation and Oxidative Damage

**Figure 4:**
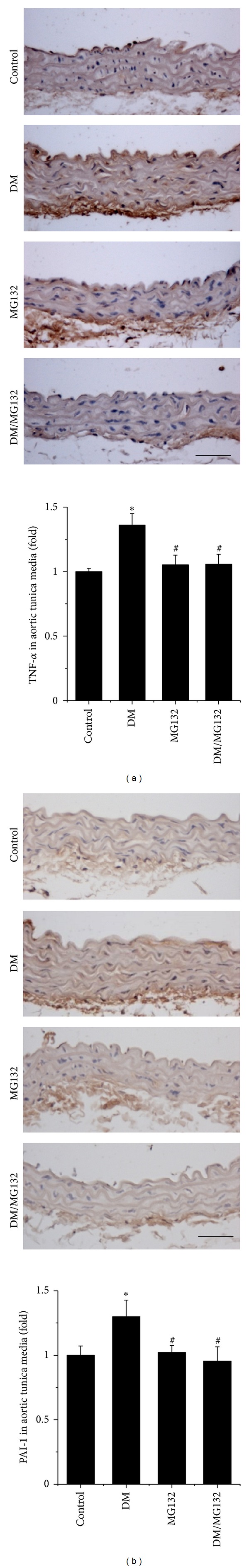
Therapeutic effect of MG132 on diabetes-induced aortic inflammation, examined by immunohistochemical staining for the expressions of TNF-*α* (a) and PAI-1 (b) followed by semi-quantitative analysis. Data were presented as means ± SD (*n* = 6). *P < 0.05 versus control;  ^#^ *P* < 0.05 versus DM. Bar = 50 *μ*M.

Immunohistochemical staining showed a significant increase in the aortic expression of inflammatory markers, TNF-*α* (Figure 4(a)) and PAI-1 (Figure 4(b)), in the aortic tunica media of DM mice at 6 months of age, which was significantly progressed compared to that of DM at 3 months of age (Figure 1(e)). However, there was no significant increase in either TNF-*α* (Figure 4(a)) or PAI-1 (Figure 4(b)) in the aorta of DM/MG132 mice.

**Figure 5:**
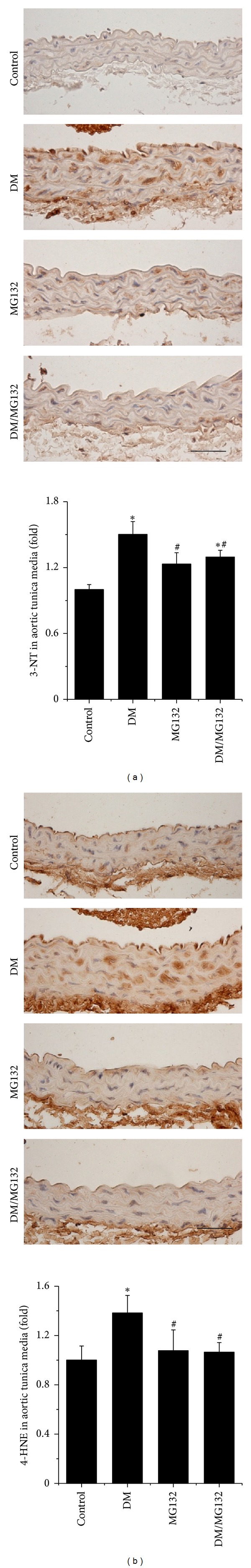
Therapeutic effect of MG132 on diabetes-induced aortic oxidative damage, examined by immunohistochemical staining for the accumulation of 3-NT (a) and 4-HNE (b) with semi-quantitative analysis. Data were presented as means ± SD (*n* = 6). *P < 0.05 versus control;  ^#^ *P* < 0.05 versus DM. Bar = 50 *μ*M.

Immunohistochemical staining also showed an increased accumulation of oxidative and nitrative damage: 3-NT (Figure 5(a)) and 4-HNE (Figure 5(b)), in aortic tunica media of DM mice, which were significantly attenuated by MG132 treatment.

### 3.4. Upregulation of Nrf2 Expression and Its Downstream Antioxidant Gene Expression by MG132 in the Aorta

**Figure 6:**
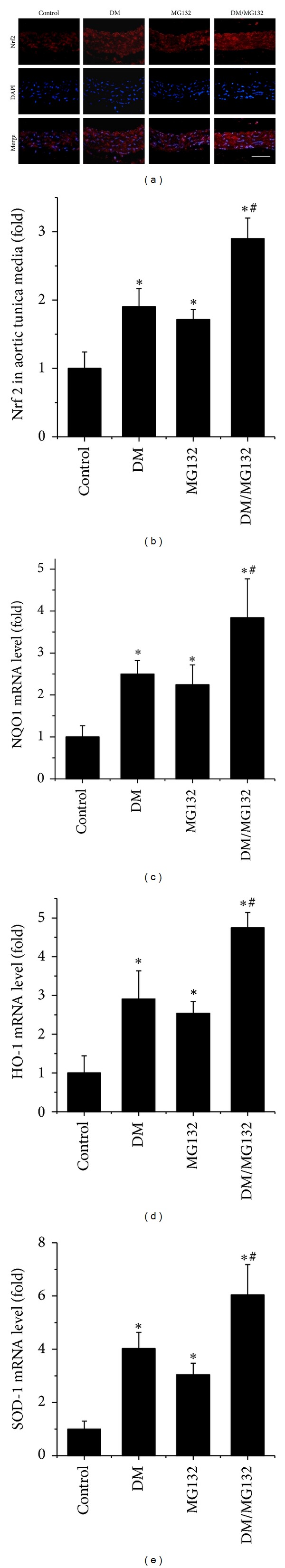
Effects of MG132 on aortic expression of Nrf2 and its downstream genes, examined by immunohistochemical staining for the expression of Nrf2 (red) (a) in aortic tunica media with semi-quantitative analysis (b). Real-time PCR was used to measure the expression of Nrf2 downstream genes at mRNA levels: NQO1 (c), HO-1 (d), and SOD-1 (e). Data were presented as means ± SD (*n* = 6). *P < 0.05 versus control;   ^#^ *P* < 0.05 versus DM. Bar = 50 *μ*M.

Since previous pathological changes may be all attributed to the increased oxidative stress, the next study is to examine the expression and transcription of Nrf2.  Figure 6(a) showed a significant increase in Nrf2 expression in the aorta of DM and MG132 mice, examined by immunofluorescent staining. There was a further increase of the Nrf2 expression in the aorta of DM/MG132 mice (Figures 6(a) and 6(b)). Furthermore, Figure 6(a) also shows an increased accumulation of Nrf2 in nuclei, suggesting the potential increase in its transcriptional function. 

**Figure 7:**
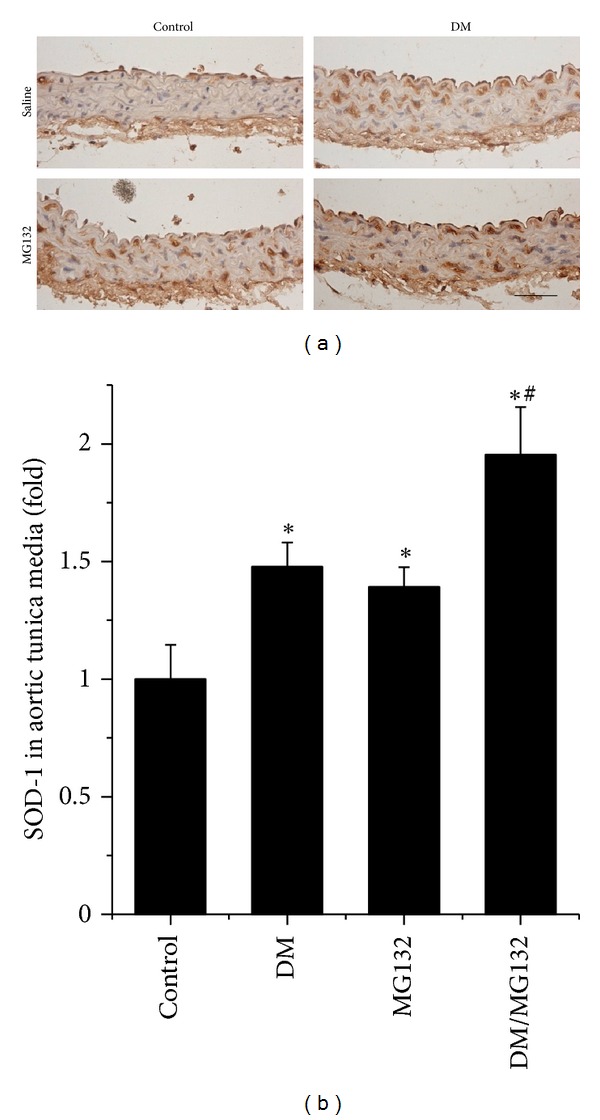
Effect of MG132 on SOD-1 expression at protein level, examined by immunohistochemical staining (a), followed by semi-quantitative analysis (b). Data were presented as means ± SD (*n* = 6). *P < 0.05 versus control;  ^#^ *P* < 0.05 versus DM. Bar = 50 *μ*M.

Analysis by qPCR revealed a significant increase in the expression of NQO1 (Figure 6(c)), HO-1 (Figure 6(d)), and SOD1 (Figure 6(e)) at mRNA level in the aorta of DM, MG132, and DM/MG132 mice with the highest expression of these endpoints in the DM/MG132 group. By immunohistochemical staining of SOD1, the upregulated mRNA expression was confirmed with the increased expression of its protein level (Figures 7(a) and 7(b)).

## 4. Discussion

The preventive effect of Nrf2 in aortic pathogeneses of several disease conditions has been appreciated; however, whether up-regulation of vascular Nrf2 can afford a therapeutic effect on oxidative stress-induced pathogenic changes in vascular system, particularly in the aorta, has not been addressed.

There were two pilot clinical trials that used the proteasomal inhibitor bardoxolone methyl (also known as CDDOMe) as a known Nrf2 activator and demonstrated certain therapeutic effects in the patients with chronic kidney diseases (CKD) and type 2 DM [22, 23]. Since in clinical study the expression of Nrf2 in the kidney of these patients was not measured, it was impossible to attribute the renal therapeutic effect of bardoxolone to Nrf2 up-regulation in these studies. In addition, there was no information regarding aortic pathological change in these studies. It is impossible to imagine whether bardoxolone induces aortic Nrf2 expression and whether aortic pathogenic changes in these diabetic patients were affected or not by bardoxolone treatment. To address such issues, animal studies have to be used.

In the study on diabetes-induced complications, streptozotocin- (STZ-) induced diabetic animals are most frequently used. However, STZ-induced diabetic animals are not perfect models for the study of diabetic complications [24] because STZ may have direct toxic effects on multiple organs [25]. OVE26 mice develop type 1 diabetes because of specific damage to *β* cells [14]. OVE26 diabetic mice have been previously used in the study of diabetes-induced cardiac and renal complications by Epstein's group [15, 26, 27]. Compared to STZ-induced diabetic mice, OVE26 mice exhibit more characteristics of human diabetic nephropathy, showing the time-dependent proteinuria [15]. One of the strengths of the present study thus is the utilization of the transgenic OVE26 diabetic mouse model.

We demonstrated here, for the first time, that there was an age-dependent development of vascular oxidative damage, inflammation, and remodeling in OVE26 type 1 diabetic mouse model, which is consistent with the observation from STZ-induced-type diabetic model [28]. More importantly we also showed that chronic treatment with low-dose MG132 can afford an effective therapy with an almost complete prevention for the progression of pathology in the aorta when the treatment was started on 3-month-old OVE26 mice that have exhibited renal dysfunction [15] and increased aortic TNF-*α* expression and 3-NT accumulation (Figure 1). The effectively therapeutic effects include the almost complete abolishment of the aortic oxidative damage, inflammation, and remodeling (Figures 2–5). We also found significant increases in Nrf2 expression and its downstream antioxidant genes, NQO1, HO-1, and SOD1, in MG132-treated control and diabetic mice (Figures 6 and 7). Therefore, we proposed that the therapeutic effect of MG132 on diabetes-induced pathological changes in the aorta may be associated with the upregulated expression of Nrf2 and its downstream antioxidant genes.

However, there may be a concern that diabetes also slightly increased the expression of Nrf2 and its downstream antioxidant genes. Why is increased pathology still observed in the aorta of diabetic mice between 3 and 6 months of age? We speculate that the increase in the expression of Nrf2 and its downstream antioxidants in the aortas of diabetic mice is an adaptive response to diabetes. Although this adaptive response is unable to provide a complete protection, it should still protect certain levels of pathogenic damage induced by diabetes; otherwise these pathogenic changes would be more severe and appear earlier. To support our assumption, an earlier study has reported that when Nrf2 gene knockout (Nrf2-KO) mice and their wild-type mice were fed a high-fat diet (HFD), HFD induced significant increases in mRNA expression of Nrf2 downstream genes in wild-type mice, but not in Nrf2-KO mice, compared with respective standard diet-fed control mice. Meanwhile, HFD-induced increases in vascular ROS levels and endothelial dysfunction were significantly more severe in Nrf2-KO than in wild-type mice. Their results suggest that adaptive activation of the endogenous Nrf2 pathway could provide certain endothelial protections under diabetic conditions, but not sufficient to completely prevent the progression of aortic pathological changes and dysfunction [11]. However, when up-regulated levels of Nrf2 and its downstream antioxidant genes in MG132-treated diabetic mice are high enough to efficiently reduce diabetes-induced oxidative damage, inflammation, and remodeling, the aortic pathogenesis might be significantly or even completely prevented, as we observed here.

Although we assume previously that Nrf2 up-regulation in the aorta in response to MG132 may be the major mechanism responsible for the aortic protection against diabetes-induced pathogenic changes, we do not exclude the possibility that MG132 may also activate other mechanisms that may also play some roles in the aortic protection against diabetes-induced pathogenic changes. It was reported that hyperglycemic elevation of NF-*κ*B-mediated renal and aortic inflammatory response in early diabetes may be related to the enhanced 26S proteasome activity, since these alterations were abolished by MG132 administration [12]. In addition, MG132 was also found to reduce oxidative stress-induced damage, which probably is related to the suppression of NF-*κ*B activation of NAD(P)H oxidase expression in coronary arterioles in type 2 diabetic mice [29]. Besides NF-*κ*B pathway, MG132 can also play a key role in anti-oxidative system by suppressing MAPK signaling pathway [30–32].

In summary, we have demonstrated here for the first time that chronic treatment with low-dose MG132 can almost completely reverse and/or prevent the progression of diabetes-induced aortic oxidative damage, inflammation, and remodeling in the transgenic OVE26 type 1 diabetic mouse model when it was given to the diabetic mice at 3 months of age, that is, at the time diabetic mice first exhibit renal dysfunction (albuminuria) and mild aortic inflammation and oxidative damage. Mechanisms responsible for the therapeutic effect of MG132 may include up-regulation of Nrf2 expression and function to afford potent antioxidant effect. Although the detailed mechanism requires additional exploration, the present study provides an interesting piece of evidence for the potential application of MG132 for diabetic patient to prevent their cardiovascular complications.
